# Clinicopathological implications of leptin and leptin receptor expression in papillary thyroid cancer

**DOI:** 10.3892/ol.2013.1125

**Published:** 2013-01-10

**Authors:** GUO-AN ZHANG, SEN HOU, SHA HAN, JIAN ZHOU, XU WANG, WEN CUI

**Affiliations:** 1 Department of Pathology, Jining Medical University, Jining, Shandong 272067;; 2 Institute of Basic Medicine, Shandong Institute of Medicine, Jinan, Shandong 250001;; 3 Life Science Experimental Center; Jining Medical University, Jining, Shandong 272067, P.R. China; 4 Center of Forensic Science, Jining Medical University, Jining, Shandong 272067, P.R. China

**Keywords:** leptin, leptin receptors, clinicopathological features, papillary thyroid cancer

## Abstract

The role of leptin and its receptors (OBRs) in the pathogenesis of various primary human malignancies has been demonstrated. However, their expression and clinicopathological significance in papillary thyroid cancer (PTC) is not fully understood. In this study, we examined the expression of leptin and OBRs in 76 PTC samples using immunohistochemistry, and their associations with clinicopathological parameters were evaluated. The expression of OBRs was observed in the tumor cell membrane and/or cytoplasm, with a positive rate of 73.7% (56/76), while leptin was expressed in the tumor cell cytoplasm in 55 of 76 cases (72.4%). The expression of either protein was associated with greater tumor size (P=0.016 for leptin and P=0.002 for OBRs). In addition, the expression levels of leptin and OBRs were associated with each other. Neither leptin nor OBR expression levels were associated with other parameters, including age, body weight, postmenopausal state, multifocality and lymph node metastasis. These data suggest that the expression of leptin and/or OBRs in PTC is associated with tumor size and may be a potential target in PTC.

## Introduction

Thyroid cancer is the most common malignancy of the endocrine system and accounts for ∼1% of all newly diagnosed cancer cases in the USA (1,2). The most frequent type is papillary thyroid cancer (PTC), which constitutes >80% of all cases. It has also been reported that the thyroid cancer incidence rate has significantly increased among males and females in numerous countries, including the USA (1), Southeast England (3), Italy (4), Lithuania (5), Canada (6) and China (7), and enhanced medical scrutiny of small tumors cannot explain this finding (1,2).

Obesity is now becoming an epidemic worldwide (8), including in China (9,10). It has been considered as a risk factor for several types of cancer, including colorectal cancer, postmenopausal breast cancer, kidney cancer and endometrial cancer (11). For thyroid cancer, a meta-analysis (12) found that a 5-kg/m^2^ increase in body mass index (BMI) was strongly associated with thyroid cancer in males (1.33, P= 0.02) and females (1.14, P=0.001), although the association is weaker in females.

One of the important mediators between obesity and increased cancer risk is leptin (11,13), which is an adipokine whose major functions are regulating appetite and energy homeostasis (14). Leptin serum levels are closely correlated with adiposity in humans. It exerts its effects through binding to its receptors (OBRs), which are located in several tissues throughout the body. Among the receptors, only the long form (OBRb) was considered to have full potential to transduce signals. The signaling pathways activated by OBRb include the classic cytokine Janus kinase 2/signal transducer and activator of transcription 3 (JAK2/STAT3) pathway; the Ras/extracellular signal-regulated kinases 1/2 (Ras/ERK1/2) signaling cascade; and the phosphoinositide 3 kinase/protein kinase B (PI3K/Akt) growth/anti-apoptotic pathway (15).

Leptin and OBRs have been reported to be overexpressed in numerous types of cancer and cancer cell lines (16). Moreover, their expression intensity is associated with cancer progression and/or prognosis in several common types of cancer, including glioblastoma (17), breast cancer (18), prostate cancer (19), ovarian cancer (20) and colorectal cancer (21,22), as revealed by immunohistochemical studies.

Leptin and OBR expression has also been studied in thyroid cancers. In a study on a large cohort of Saudi PTC patients (23), OBRs and leptin were found to be expressed in 80.1% (410/512) and 49.1% (252/513) of PTC patients, respectively, and OBR expression was strongly associated with age, gender, extrathyroidal extension, tumor stage, tumor size, node metastasis and histological type. However, in another study of Chinese PTC patients in Taiwan (24), different results were obtained. OBRs and leptin were detected in 51.0% (25 of 49) and 36.7% (18 of 49) of cases, respectively, and neither were associated with age, gender, extrathyroidal extension, multifocality, thyroiditis or BMI. The exception was tumor size, which was shown to be associated with OBR and leptin expression. The study also revealed that PTC tumors with both leptin and OBR expression were more likely to develop lymph node metastasis compared with tumors with neither leptin nor OBR expression. However, reasons for the differences between the results of the two studies are unknown.

The aim of the present study was to detect the expression of leptin and OBRs in a group of Chinese mainland PTC patients, and to determine whether their expression correlated with patient and tumor characteristics.

## Patients and methods

### Patients

This study included 76 patients who underwent thyroidectomy in Jining First People’s Hospital between 2010 and 2011. Clinical and histopathological data, including tumor size, multifocality, lymph node metastasis, age and weight, were reviewed for all patients. Hematoxylin and eosin (HE)-stained slides for each patient were reviewed to confirm the diagnosis of PTC. All patients were euthyroid prior to surgery. The ethics committee of the Affiliated Hospital of Jining Medical College censored and approved the study. Written informed consent was obtained from the patients.

### Immunohistochemistry (IHC)

For immunohistochemical staining, 5-mm sections of formalin-fixed, paraffin-embedded tissue blocks were dewaxed in xylene and rehydrated through graded alcohol and phosphate-buffered saline (PBS). Antigen retrieval was conducted by boiling slides in 10 mM sodium citrate buffer (pH 6.0) for 10 min. The sections were then treated with 0.3% hydrogen peroxide at room temperature for 10 min to quench endogenous peroxidase activity. After rinsing in PBS, the sections were blocked with 10% normal rabbit serum (for OBRs) or goat serum (for leptin) at 37°C for 1 h. Then the sections were incubated overnight at 4°C in humid chambers with primary antibody. The primary polyclonal rabbit anti-leptin antibody (A-20, Santa Cruz Biotechnology, Inc., Santa Cruz, CA, USA) was diluted at 1:200 and the primary polyclonal goat anti-OBR antibody (M-18, for all forms of OBRs; Santa Cruz Biotechnology, Inc.) was diluted at 1:200. Subsequently, the sections were incubated with a polyperoxidase-conjugated anti-rabbit or anti-goat secondary antibody (Gold Bridge, Beijing, China) for 30 min. Following a PBS wash, DAB substrate (Gold Bridge) was added to the sections for 30 min. Finally, the slides were counterstained with hematoxylin, dehydrated after a standard procedure and sealed with coverslips. Sections that had been incubated with PBS instead of primary antibody were used as a negative control.

### Evaluation of immunostaining

The expression levels of leptin and OBRs were evaluated semiquantitatively by two experienced pathologists (X.W. andW.C.). Scoring was based on staining intensity and staining extent. Staining intensity was scored as 0 (negative), 1 (weak), 2 (moderate) or 3 (strong). Staining extent was scored as 0 (0%), 1 (1–25%), 2 (26–50%) or 3 (51–100%) according to the percentage of positively stained cells. Multiplied scores of intensity and extent were used as the final staining score. Patients were sorted into 2 groups; positive expression was defined by final staining scores of 6 and 9, whereas the remaining cases (final scores 0–4) were classified as negative expression.

### Statistical analysis

The correlation between the expression of OBRs/leptin and clinicopathological features was analyzed. Continuous variables are expressed as the mean ± standard deviation (SD). Data were evaluated for significant differences by the 2-tailed Student’s t-test or the χ^2^ test using the Statistical Package for Social Sciences, version 13.0 (SPSS, Inc., Chicago, IL, USA). P<0.05 was considered to indicate statistically significant differences.

## Results

### Immunohistochemical detection of OBRs and leptin

**Figure 1:**
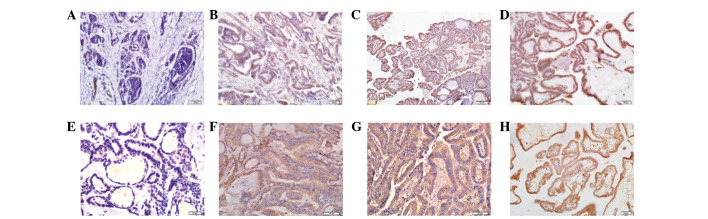
Leptin and OBR expression in PTC samples. (A) Negative, (B) weak, (C) moderate and (D) strong expression of leptin in PTC samples is shown in the first row. The second row demonstrates expression of OBRs with (E) negative, (F) weak, (G) moderate and (H) strong staining. OBR, leptin receptor; PTC, papillary thyroid cancer.

Expression levels of OBRs and leptin were determined by IHC in 76 PTC samples. OBR expression was observed in tumor cell membrane and/or cytoplasm with a positive rate of 73.7% (56/76), while leptin was expressed in tumor cell cytoplasm in 55 of 76 cases (72.4%; Fig. 1). In 57.9% (44/76) of cases, both leptin and OBRs were expressed. The expression levels of OBRs and leptin in PTC samples were significantly associated with each other, as shown in Table I (P<0.05).

### Association of expression of leptin and OBRs with clinicopathological parameters

Our study on the association of OBR and leptin expression with clinicopathological data demonstrated that PTC samples with positive staining for OBRs (P=0.002) or leptin (P=0.016) were associated with a larger tumor size. Neither leptin nor OBR expression was associated with other parameters, including age, body weight, postmenopausal state, multifocality or lymph node metastasis (Table II).

## Discussion

Our study showed that 73.9% (56/76) and 72.4% (55/76) of PTC samples expressed OBRs and leptin, respectively. These data are different from those of Cheng *et al*(24) who revealed that OBRs and leptin are expressed in 51.0% (25/49) and 36.7% (18/49) of PTC cases, respectively, and Uddin *et al*(23) whose results showed that OBRs and leptin are expressed in 80.1% (410/512) and 49.1% (252/513) of PTC cases, respectively. Although the positive rates are different, all three studies indicated that a proportion of PTC tumors express leptin and OBRs. However, the reasons for differences between the data requires further study, particularly the difference between the data of this study and those of Cheng *et al*, due to the similar ethnicity of the PTC patients and also since the same antibodies were used.

In this study, 57.9% of PTC cases expressed both leptin and OBRs, and it was also observed that the expression levels of leptin and OBRs were significantly associated with each other. Similar results were obtained by Cheng *et al*(24) in PTC and in other cancer types, including endometrial cancer (25), colorectal cancer (26) and breast cancer (18,27). This suggests that leptin and OBR expression may be induced by some of the same mechanisms, or that the expression of one molecule may be induced by the other. Indeed, leptin and OBR expression is correlated with HIF-1α in endometrial (25) and colorectal cancer (26), which suggests that HIF-1α may induce both. More direct evidence from *in vitro* cell line studies indicated that IGF-1, insulin and estradiol induced the expression of leptin and OBR mRNA in the MCF-7 breast cancer cell line, while in MDA-MB-231 cells, leptin and OBR mRNA expression was induced by insulin or hypoxia (28). There is also evidence demonstrating that leptin enhanced the expression of OBRs, for example in ZR-75-1 breast cancer cells (29). Whether or not these mechanisms exist in PTC cells is largely unknown. However, insulin was previously shown to upregulate OBR expression in a time- and dose-dependent manner, while the hypoxia-mimicking agent cobalt chloride had no effect on OBR expression in PTC cell lines (30). The effect of insulin on leptin expression in thyroid cancer has not been studied.

In the present study, leptin and OBR expression levels were found to be associated with PTC tumor size, which is similar to the observations of two previous studies (23,24). This result is to be expected, considering that leptin, through OBRs, has been shown to promote proliferation and inhibit apoptosis in numerous types of cancer (16) and PTC cell lines (23,31,32).

We have identified several weaknesses in this study. Firstly, the small number of patients enrolled means there is a higher chance of producing imprecise results, compared with a larger group of patients. The second and more important point is the lack of study of the survival rate or recurrence in these patients due to the relatively benign nature of PTC. However, Uddin *et al*(23) showed that patients with overexpression of OBRs had a reduced disease-free survival rate of 68.9% at 5 years, compared with 79.3% with reduced OBR expression.

In summary, our observations have added to the evidence that leptin and OBRs are expressed in PTC and their expression is associated with each other and with PTC tumor size. Further study is required to determine the potential prognostic and therapeutic implications of the leptin/OBR system.

## Figures and Tables

**Table I:** Correlation between OBR and leptin expression.

OBR expression	Leptin expression			
	Positive	Negative	Total	P-value
Positive	44	12	56	
Negative	11	9	20	
Total	55	21	76	P<0.05

OBR, leptin receptor.

**Table II:** Expression of leptin and OBRs relative to the clinicopathological characteristics of PTC.

	Leptin expression			OBR expression		
Characteristic	Positive	Negative	P-value	Positive	Negative	P-value
No. of patients	55	21		56	20	
Age (years), mean ± SD	46.0±13.3	53.4±12.3	0.878	47.7±14.7	48.9±8.9	0.736
Body weight (kg), mean ± SD	66.5±10.8	68.4±11.9	0.427	67.2±12	66.2±8.5	0.703
No. of postmenopausal patients (56 female patients) (%)	14 (33.3)	6 (42.9)	0.520	15 (35.7)	5 (35.7)	1.000
Lymph node metastasis (%)	17 (30.9)	9 (42.9)	0.326	20 (35.7)	6 (30)	0.644
Tumor size (mm), mean ± SD	22.2±10.3	17.1±12.8	0.016	23.7±10.1	15.1±10.3	0.002
Multifocality (%)	31 (56.4)	14 (66.7)	0.414	32 (57.1)	13 (65.0)	0.539

OBR, leptin receptor; PTC, papillary thyroid cancer; SD, standard deviation.
